# Congenital Hyperinsulinism: Diagnosis and Treatment Update

**DOI:** 10.4274/jcrpe.2017.S007

**Published:** 2017-12-30

**Authors:** Hüseyin Demirbilek, Khalid Hussain

**Affiliations:** 1 Hacettepe University Faculty of Medicine, Department of Paediatric Endocrinology, Ankara, Turkey; 2 Sidra Medical and Research Center, Clinic of Paediatric Medicine, Doha, Qatar

**Keywords:** Hyperinsulinaemic hypoglycaemia, congenital hyperinsulinaemia, children, diffuse congenital hyperinsulinism, focal congenital hyperinsulinism, sirolimus

## Abstract

Pancreatic β-cells are finely tuned to secrete insulin so that plasma glucose levels are maintained within a narrow physiological range (3.5-5.5 mmol/L). Hyperinsulinaemic hypoglycaemia (HH) is the inappropriate secretion of insulin in the presence of low plasma glucose levels and leads to severe and persistent hypoglycaemia in neonates and children. Mutations in 12 different key genes (ABCC8, KCNJ11, GLUD1, GCK, HADH, SLC16A1, UCP2, HNF4A, HNF1A, HK1, PGM1 and PMM2) that are involved in the regulation of insulin secretion from pancreatic β-cells have been described to be responsible for the underlying molecular mechanisms leading to congenital HH. In HH due to the inhibitory effect of insulin on lipolysis and ketogenesis there is suppressed ketone body formation in the presence of hypoglycaemia thus leading to increased risk of hypoglycaemic brain injury. Therefore, a prompt diagnosis and immediate management of HH is essential to avoid hypoglycaemic brain injury and long-term neurological complications in children. Advances in molecular genetics, imaging techniques (^18^F-DOPA positron emission tomography/computed tomography scanning), medical therapy and surgical advances (laparoscopic and open pancreatectomy) have changed the management and improved the outcome of patients with HH. This review article provides an overview to the background, clinical presentation, diagnosis, molecular genetics and therapy in children with different forms of HH.

## INTRODUCTION

Hyperinsulinaemic hypoglycaemia (HH), refers to a clinically, genetically and morphologically heterogeneous group of disorders associated with dysregulated insulin secretion. It is the most common cause of persistent hypoketotic hypoglycaemia in neonates and infants and is associated with a significant risk of permanent brain damage. Therefore, it is essential to make a prompt diagnosis and institute immediate management to prevent complications such as epilepsy, cerebral palsy and neurodevelopemental deficits (1).

The metabolic action of insulin on glucose and fuel metabolism increases the risk of neurological injury. Insulin decreases blood glucose level by increasing its peripheral consumption, stimulates glycogen synthesis and inhibits glycogenolysis and gluconeogenesis. On the other hand, insulin has an anabolic effect on fat tissues. It stimulates lipogenesis, inhibits free fatty acid release, and their beta-oxidation and thus inhibits ketone body formation. This accounts for the hypoketotic state, decreasing the availability of alternative fuels for cerebral metabolism (2). As the brain of neonates and infants has a higher rate of glucose comsumption compared to adult subjects, it is more vulnerable to hypoglycaemic brain injury. HH typically presents in the newborn period with severe hypoglycaemia but can also present in infancy, childhood and even as late as adulthood with variable severity and etiology (3,4).

HH can be transient due to certain risk factors, such as birth asphyxia, intra-uterine growth retardation, maternal diabetes mellitus (5), or associated with various overgrowth syndromes like Beckwith-Wiedemann syndrome or metabolic conditions such as congenital disorders of glycosylation (6). Genetic forms of HH congenital hyperinsulinism (CHI) are due to mutation in the genes involved in the regulation of insulin secretion. HH typically presents with fasting hypoglycemia but can present with postprandial hypoglycaemia or in some cases hypoglycaemia can be provoked by protein/leucine loading or even exercise. Patients with HH can vary in their presentation from having no symptoms to having severe, medically unresponsive disease which might require a near total pancreatectomy (7).

Histologically, CHI is classified into three subgroups: diffuse, focal and atypical forms (8,9). Diffuse disease affects all the islets in the pancreas, whereas in focal disease the abnormality is confined to a small region of the pancreas. Atypical histological forms of CHI have recently been described (10). Although all the histological subtypes are clinically and biochemically indistinguishable, their differentiation at the histological level is important from the point of the view of management. Recent advances in imaging techniques using ^18^F-fluoro-L-dihydroxyphenylalanine (^18^F-DOPA) positron emission tomography/computed tomography (PET/CT) have fundamentally changed management strategies, particularly in patients with focal CHI (11,12).

Mutations in key genes which are involved in the regulation of insulin secretion from pancreatic β-cells underlie the molecular basis of CHI. Until recently mutations in only 12 different genes (ABCC8, KCNJ11, GLUD1, GCK, HADH, SLC16A1, HNF4A, HNF1A, HK1, PGM1 and PMM2) that lead to dysregulated secretion of insulin had been described (3,13,14,15,16,17,18). More recently there have been single case reports of potentially novel genetic mechanisms of HH associated with other syndromic features (19,20). In the vast majority of patients who are diazoxide responsive, the genetic basis of HH is still not known. This review aims to give an overview of the biochemical and molecular basis of CHI with a focus on describing the latest advances in the diagnosis and treatment of this complex condition.

### Physiological Mechanisms Regulating Insulin Secretion from Pancreatic β-cells in Congenital Hyperinsulinism

During the intrauterine period the fetus receives glucose across the placenta via facilitated diffusion. After birth, in term healthy newborns with no risk factors for hypoglycemia, plasma glucose levels tend to shows a sharp decline during the first 24-48 hours, but will then normalize to values around 3.5-5.5 mmol/L. This maintenance of a normal plasma glucose concentration requires an adequate supply of exogenous glucose, endogenous fat, glycogen and potential gluconeogenic substrates (e.g. amino acids, glycerol and lactate). In addition, a functional endocrine system that integrates and modulates substrate mobilization, interconversion and utilization is important, as are the key enzymes involved in glycogen synthesis/glycogenolysis, glycolysis, gluconeogenesis, lipolysis and ketogenesis.

The pancreatic β-cells possesses a signal transduction system, whereby fuel metabolism is intricately linked to regulated insulin secretion (21). Glucose is the most important fuel involved in this so called stimulus-response coupling mechanism. This stimulus response-coupling event is controlled by adenosine triphosphate (ATP)-sensitive potassium channels (K_ATP_) located in the pancreatic β-cells membrane (22). Glucose enters the β-cells through facilitative glucose transporters, particularly glucose transporter 2 (GLUT 2) and is converted to glucose-6-phosphate by the enzyme glucokinase (GCK) (23). GLUT 2 has high affinity for glucose which allows glucose transport in proportion to the plasma glucose concentration (24).

**Figure 1:**
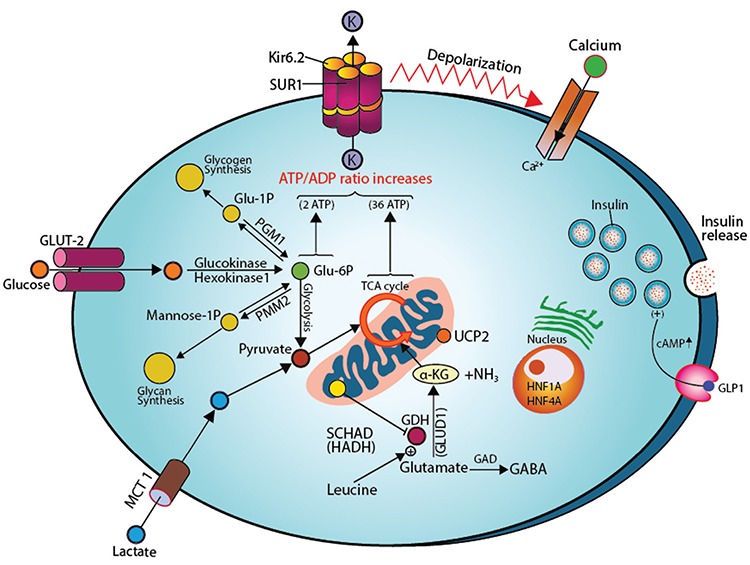
Regulation of insulin release from pancreatic β-cell and sites of gene mutations involved in the genetics etiology of hyperinsulinaemic hypoglycaemia SUR1: sulphonlyurea receptor 1, Kir6.2: inwardly rectifying potassium channel 6.2, K: potassium, MCT1: monocarboxylate transporter 1, Glu: glucose, P: phosphorus; PGM1: phosphoglucomutase 1, PMM2: phosphomannose-mutase 2, UCP2: mitochondrial uncoupling protein 2, NH3: ammonia, GDH: glutamate dehydrogenase, GLUD1: glutamate dehydrogenase 1 gene, SCHAD: short-chain L-3-hydroxyacyl-CoA dehydrogenase, HADH: hydroxy-acyl-CoA dehydrogenase, HNF1A and 4A: hepatocyte nuclear factor 1A and 4A, Ca+2: calcium; GAD: glutamate decarboxylase enzyme, GABA: γ-aminobutyric acid, GLP1: glucagon like peptide 1, cAMP: cyclic adenosine monophosphate (amplifier for the exocytosis of insulin secreting granule)

Glycolysis generates high energy molecules such as ATP and this leads to an increase in the ratio of ATP/adenosine diphosphate (ADP) resulting in the closure of the ATP-K_ATP_. The inwardly rectifying potassium (Kir6.2) subunit of the K_ATP_ channels are responsible for trafficking of intracellular and extracellular ion exchange, thus maintaining a steady state membrane potential. The closure of the K_ATP_ channels results in depolarization of pancreatic β-cells membranes and activation of intramembraneous voltage-gated calcium channels. Calcium enters into β-cells through these voltage-gated calcium channels and an increase in intracellular calcium triggers secretory granule exocytosis and insulin release (Figure 1).

GCK plays a critical role in acting as a gluco-sensor, providing a link between the extracellular plasma glucose concentration and the metabolism of glucose in β-cells (25). When the plasma glucose concentration is increased, the activity of GCK is also increased, hence increasing insulin secretion from the β-cells (Figure 1). Conversely, as the plasma glucose concentration decreases, insulin secretion decreases and serum insulin becomes undetectable when the plasma glucose level is below 3 mmol/L (26,27).

### Clinical Presentation and Biochemical Diagnosis of Hyperinsulinaemic Hypoglycaemia

Patients with HH can present with a wide range of symptoms ranging from non-specific adrenergic symptoms (poor feeding, hunger, palpitations, sweating) to life-threatening, neuroglycopenic symptoms (seizures, unconsciousness, lethargy, coma and even death) arising from an inadequate supply of glucose to the brain, resulting in impairment of brain function.

HH most commonly presents during the neonatal period, but can also present during infancy, childhood and even adulthood (4,28). The clinical presentation of hypoglycaemia is most severe in the newborn and may be quite subtle in infancy and the childhood period. Therefore the diagnosis might be missed until later in life (29,30,31). There can be marked phenotypical variability even within the same family.

Newborns with HH may be macrosomic due to intrauterine hyperinsulinaemia. However, the absence of macrosomia does not exclude HH. Hypertrophic cardiomyopathy and hepatomegaly (increased storage of glucose as glycogen) are observed in some patients with HH. The mechanism of cardiomyopathy and hepatomegaly in these patients is unclear but might be related to the effect of foetal hyperinsulinaemia (1).

Early diagnosis of HH is fundamentally important in preventing hypoglycaemic brain injury. Hence, clinicians should always be aware of recognising HH and managing these patients. In any patient with recurrent or persistent hypoglycaemia, HH should be suspected and critical samples at the time of hypoglycaemic episodes should be collected. An intravenous glucose infusion rate requirement of >8 mg/kg/min (normally is 4-6 mg/kg/min) is virtually diagnostic of HH (1). In milder forms of HH, it will be important to establish the duration of fasting and whether the hypoglycaemia is precipitated by meals (protein sensitivity) or by exercise.

Biochemically in HH, there is an inappropriate concentration of serum insulin/c-peptide for the level of plasma glucose (spontaneous or provoked). Low or undetectable serum insulin levels during hypoglycaemia do not exclude the diagnosis of HH (29,30). In some cases serum C-peptide levels (≥0.5 ng/mL) and IGFBP-1 (≤110 ng/mL) may help confirm the diagnosis of HH with specificities of 100% and 96.6%, respectively (29). The metabolic effect of inappropriate insulin secretion is reflected by inappropriately low levels of serum ketone bodies and fatty acids during hypoglycaemic episodes. There is no correlation between measured serum insulin concentration and the severity of the hypoglycaemia (31). In some difficult cases, the diagnosis of HH should not be based on an isolated serum insulin/c-peptide concentration but on the clinical presentation and the biochemical profiles of insulin action (low β-hydroxybutyrate and fatty acid concentrations). The diagnostic criteria for HH are summarized in Table 1 (29,32,33).

In some instances certain biochemical and clinical features may help in the diagnosis of specific forms of CHI. An elevated serum ammonia concentration in a patient with HH is suggestive of the hyperinsulinism and hyperammonaemia (HI/HA) syndrome (34). Raised plasma hydroxybutyrylcarnitine and urinary 3-hydroxyglutarate are diagnostic of a rare type of congenital HH [hydroxyacyl-Coenzyme A dehydrogenase (HADH) deficiency] (35).

Some types of HH are elicited only after provocation testing. For example in patients who have the HI/HA syndrome and HADH, protein/leucine loading precipitates hypoglycaemia (36). Patients with exercise-induced HH will require a formal exercise test and or a pyruvate load to demonstrate post-exercise induced HH (37,38). In some patients, a positive glycaemic response (rise in the plasma glucose concentration of >1.5 mmol/L from baseline) following an intramuscular/intravenous injection of glucagon at the time of hypoglycaemia provides supportive evidence (39). A glycaemic response to a subcutaneous dose of octreotide may also aid diagnosis, along with decreased serum levels of insulin growth factor binding protein-1 (IGFBP-1) as insulin suppresses the transcription of the IGFBP-1 gene (40).

### Transient Forms of Hyperinsulinaemic Hypoglycaemia

There is no precise definition of transient HH, but if the hypoglycaemia resolves spontaneously within a few days (or up to a week) then it might be considered to be transient. Transient HH typically develops in newborns with certain risk factors [such as maternal diabetes mellitus, the use of intravenous dextrose given during labour, intrauterine growth restriction (IUGR), and perinatal asphyxia (Table 1)]. Some newborns with IUGR and asphyxia have a severe and protracted form of HH which requires treatment with diazoxide (41). The underlying molecular mechanisms in transient cases are not known, but some cases are due to mutations in HNF4A and HNF1A (33). In addition, transient HH has been described in some newborns with no underlying risk factors (42).

### Genetic Forms of Hyperinsulinaemic Hypoglycaemia

The genetic basis of CHI involves defects in genes that encode key proteins involved in the regulation of insulin release from the pancreatic β-cell. These defects lead to disturbances in glucose-stimulated insulin secretion and inappropriate release of insulin from pancreatic β-cells. Currently, mutations in 12 genes have been reported to cause CHI and more recently there have been isolated case reports of potential novel genetic mechanisms in patients with CHI and other syndromic features. The underlying molecular mechanisms that causes CHI in the vast majority of patients who are diazoxide responsive are still unknown. Table 2 lists the transient and persistent causes of HH.

### Genetics of Hyperinsulinaemic Hypoglycaemia

**a) Pancreatic β-cell KATP Channel Defects**

K_ATP_ channels are located in the β-cell membrane and transduce the metabolic signals generated by glucose metabolism to regulate insulin secretion (33). The K_ATP_ channel complex is composed of four outer, sulphonlyurea receptor 1 (SUR1) subunits that are encoded by the ATP Binding Cassette Subfamily C Member 8 (ABCC8) gene and the four inner, pore-making, Kir6.2 channel proteins, encoded by the Potassium Voltage-Gated Channel Subfamily J Member 11 (KCNJ11). Both these genes are located on chrosome 11p15.1. The SUR1 component regulates the activity of the Kir6.2 proteins and functions as the binding site for the K_ATP_ channel opener (diazoxide) and sulphonylureas (43,44). The inner Kir6.2 protein forms a pore allowing potassium influx across the β-cell membrane. A change in the ratio of ATP to ADP causes closure of the K_ATP_ channel and triggers depolarisation of the cell membrane, activating the voltage-gated calcium channels (45). This in turn causes insulin release through exocytosis (46,47,48).

Mutations in the genes encoding K_ATP_ channel proteins are the most common cause of severe CHI (49,50). Recessive inactivating (or loss-of-function) K_ATP_ channel gene mutations predominantly cause medically unresponsive diffuse CHI (4,6,51,52). These mutations can either inhibit the trafficking of channel proteins (SUR1) to the plasma membrane or channel activity (33). Autosomal dominantly inherited mutations usually cause milder forms of CHI (53,54). Recently a novel phenomenon, describing the combination of heterozygous mutations in the ABCC8 and KCNJ11 genes has been described (55).

**b) Glutamate Dehydrogenase (GDH) and Hyperinsulinaemia-hyperammonaemia Syndrome (HI/HA)**

The glutamate dehydrogenase 1 (GLUD1) gene encodes for the mitochondrial matrix enzyme, GDH which catalyzes the oxidative deamination of glutamate to α-ketoglutarate and ammonia (56). GDH is allosterically activated by the amino acid leucine and inhibited by guanosine-5’-triphosphate (GTP) (57). GLUD1 mutations decrease the sensitivity of the allosteric inhibitor, GTP, thereby resulting in a gain-of-function of the GDH enzyme. Dominantly inherited GLUD1 mutations are associated with fasting and leucine (protein) induced postprandial HH, with elevated plasma ammonia (also known as HI/HA syndrome) concentration. Interestingly in a mutant GDH mouse model carrying the H454Y mutation, in addition to the loss of GTP inhibition on GDH activity, there was also inhibition of glucagon secretion (58). This inhibition of glucagon secretion may also contribute to the development symptomatic hypoglycemia in these patients (58).

GLUD1 mutations are the second most common cause of CHI. Studies to date have identified mutations in exons 6, 7, 11 and 12 and 13 (34,59). Although GLUD1 activating mutations are mostly de novo, autosomal dominant forms have also been reported (59,60). HI/HA syndrome patients are diazoxide responsive and in some cases dietary protein restriction might be necessary. Patients with GLUD1 mutations have been reported to develop epileptic seizures regardless of the severity and frequency of hypoglycaemic episodes. Urinary α-ketoglutarate is elevated in patients with HI/HA syndrome (61).

**c) Mutations in Mitochondrial L-3-Hydroxyacyl-CoA Dehydrogenase (HADH) and CHI**

HADH or short chain L-3-Hydroxyacyl-CoA dehydrogenase is another mitochondrial enzyme that is involved in the penultimate step of β-oxidation of fatty acids. This gene is most abundantly expressed in pacreatic islet cells, while also present in other extrapancreatic tissues such as the liver, kidneys, muscle and heart (62). The HADH gene has 8 exonic regions and autosomal recessive loss-of-function mutations impair the enzymatic inhibitory effect of HADH on GDH (63,64,65). This in turn causes a rise in incracellular ATP and inappropriate -leucine sensitive- HH. These observations suggest that GDH plays a pivotal role in fatty acid and amino acid metabolism to control insulin secretion (33). The serum ammonia level is normal in these patients. HADH gene mutations can lead either to severe neonatal HH or to mild, late (even adult) onset, protein-induced HH (66,^67^). Mutations of HADH gene have been reported as one of the most common cause of diazoxide responsive CHI in consanguineous pedigrees. Therefore, HADH sequence analysis is recommended for all patients with diazoxide-responsive HH when recessive inheritance is suspected (68). Patient with HADH mutations may have elevated plasma concentrations of 3-hydroxy-butyryl-carnitine and urinary 3-hydroxy-glutaric acid (35,65).

**d) Activating Mutations in GCK and CHI**

GCK catalyses glucose to glucose-6-phosphate conversion as substrate for the glycolytic pathway leading to ATP generation and glucose-dependent insulin release. GCK has high affinity for glucose, serving as a glucose-sensor in pancreatic β-cells. The GCK gene has 12 exons and encodes the enzyme, GCK. GCK can be found in the pancreatic β-cells, liver and brain (69). Dominant activating mutations in GCK cause alteration in both protein structure and function. The affinity of mutated GCK enzyme for glucose increases, thereby the threshold for glucose-stimulated insulin release is decreased (70,71). Patients with GCK mutations can have a wide range of clinical presentations. These vary from severe, neonatal-onset HH which is medically unresponsive and requiring surgery to mild, adult-onset HH which may be asymptomatic (3,72,73,74,75).

**e) Mutations in Solute Carrier Family 16 Member 1 (SLC16A1) and Exercise-induced CHI**

Monocarboxylate transporter 1 (MCT1) protein, encoded by the SLC16A1 gene, is involved in the transport of pyruvate and lactate across the β-cell membrane. These monocarboxylates (pyruvate and lactate) serve as substrates for the Krebs cycle. Under physiological conditions the SLC16A1 gene is silenced in pancreatic β-cells suggesting that both pyruvate and lactate are prevented from stimulating insulin secretion (33). Dominant gain-of-function mutations in the promotor region of SLC16A1 cause increased expression of MCT1 in β-cells. This in turn leads to glycolysis-generated pyruvate to continually enter the Kreb’s cycle and stimulate insulin secretion in states of low plasma glucose during anaerobic exercise, and in particular strenuous exercise (76). A pyruvate load or excercise test may precipitate HH and may be used for diagnostic purposes (38). These patients are often diazoxide responsive and avoiding strenuous exercise is advised (37).

**f) Hepatocyte Nuclear Factor (HNF) 1A&4A (HNF1A&4A) and CHI**

The HNFs, HNF1-a and HNF4-a, are transcription factors for nuclear hormone receptors expressed in pancreatic β-cells and regulate glucose-dependent insulin secretion (77,78). The hepatocyte nuclear factors 1A and 4A genes (HNF1A/HNF4A) encode for the HNF1-a and HNF4-a proteins, respectively. Heterozygous loss-of-function mutations in HNF4A and HNF1A lead to HH in the newborn period and maturity onset-diabetes (type 1 and 3) later in life (79,80,81,82). CHI due to mutations in both HNF1A and HNF4A are characterized by macrosomic birth and mild transient to severe diazoxide-responsive HH (6,13,52,79,83,84,85). CHI due to HNF4A gene has been reported with increased levels of glycogen in erythrocytes, elevated liver transaminases and increased echogenicity on liver ultrasonography, suggesting a glycogenosis-like phenotype (86,87). In some patients with diazoxide responsive HH, mutations in HNF1A and HNF4A may be common (85,88).

**g) Mutations in the Mitochondrial Uncoupling Protein 2 (UCP2) and CHI**

UCP2, an inner mitochondrial carrier protein which encoded by the UCP2 gene, is widely expressed in tissues, including pancreatic islets (89,90). UCP2 mediates proton leak across the inner mitochondrial membrane, thereby inhibiting ATP generation through mitochondrial oxidative metabolism and negatively regulates glucose mediated insulin secretion (90,91). Inactivating heterozygous mutations of the UCP2 gene would therefore, enhance glucose oxidation and increase intracellular ATP synthesis leading to HH (90,92). CHI due to UCP2 mutations can present with a clinical phenotype ranging from transient HH to prolonged HH (28,90,93). In one study UCP2 variants were found in 2.4% from a cohort of 211 diazoxide responsive patients (28). However, in a more recent study, no protein truncated variants were detected in the UCP2 gene among 206 diazoxide responsive patients (94). The only variant detected was considered to be a common polymorphism. This suggests, therefore, that the role UCP2 in CHI needs further investigation.

**h) Somatic overexpression of Hexokinase 1 (HK1) and CHI**

HK1 is located on chromosome 10 and encodes the enzyme; HK1. Hexokinases are a group of enzymes that catalyse the first step of glucose metabolism, of which HK1 is the predominant enzyme. It catalyses the phosphorylation of glucose to produce glucose-6-phosphate as substrate for glycolysis. Normally, HK1 expression is silenced in the pancreatic β-cells. Recently however, a report identified a dominant gain-of-function mutation in the HK1 gene in a family with “idiopathic hypoglycaemia of infancy” (17). Further evidence for the role of overexpression of HK1 has been reported in an in vitro study evaluating pancreatic specimens of five CHI cases which showed inappropriate expression of “HK1” in a subset of pancreatic β-cells. In these pancreatic specimens the K_ATP_ channel was functional but there was inappropriate insulin secretion at low plasma glucose levels (1 mmo/L) (95).

**i) Phosphoglucomutase 1 (PGM1) Gene Mutations and CHI**

PGM1 catalyses the reversible conversion of glucose-6-phosphate to glucose-1-phosphate involved in glycogen metabolism. Recently, a recessive loss-of-function mutation in the PGM1 gene that encodes the enzyme PGM1 has been shown to be associated with hypoglycaemia, similar to glycogenosis (18). Patients with these inactivating mutations have an exaggerated glucose-mediated insulin secretion and therefore present with fasting hyperketotic hypoglycaemia, as well as postprandial HH (15).

**j) Phosphomannomutase 2 (PMM2) Gene Mutations and CHI**

The enzyme PMM2 is involved in glycosylation and the PMM2 gene has recently been reported to cause HH as well as congenital polycystic kidney disease in 17 children from 11 unrelated families (16). The group reported a promoter mutation (c.-167G>T) in the PMM2 gene in all affected patients. This mutation has been shown to alter insulin secretion from pancreatic β-cells.

**k) Mutations in CACNA1D and CHI (Single Case Report)**

CACNA1D encodes an L-type voltage-gated calcium channel that plays a pivotal role in the regulation of insulin secretion from pancreatic β-cells. A patient with a CACNA1D gene mutation has been reported with HH, heart defects and severe hypotonia (20) but the molecular mechanism leading to HH is still not clear.

**l) Mutations in Forkhead Box Protein A2 (FOXA2) and CHI (Single Case Report)**

A case has been reported of a mutation in FOXA2 with hypopituitarism, HH and endoderm-derived organ abnormalities (19). Again the moleclar basis of the HH observed in the patient was not elucidated.

### Hyperinsulinaemic Hypoglycaemia Management

The cornerstone of clinical management involves the early diagnosis and starting of appropriate therapy for patients with all forms of HH. The aim is to keep plasma glucose levels above 3.5 mmol/L given that the brain is deprived of alternative substrates. The treatment options includes medical, surgical or sometimes combination therapies.

### Emergency Management

**Parenteral glucose infusion:** If the patient is unable to take an oral feed then 2 mls/kg of 10% glucose should be administered intravenously as a bolus. In some instances, a repeat bolus may be required, but further repeated boluses should be avoided, as the bolus of glucose is a potent trigger for insulin secretion. Normoglycemia should be achieved by delivering a continuous intravenous glucose infusion starting with 6-8 mg/kg/min. Patients with HH may require >25 mg/kg/min of intravenous glucose infusion to maintain normoglycaemia.

**Glucagon administration:** Glucagon is a key counter-regulatory hormone and is used as a first line therapy for managing CHI patients, particularly in emergency situations where patients are unable to take oral feed and/or intravenous access is difficult to obtain (32,96). Glucagon, in the short-term, induces glycogenolysis, gluconeogenesis and lipolysis and causes a rapid increase in plasma glucose within a few minutes after administration. The recommended single dose is 0.5-1 mg via intramuscular or subcutaneous injection. Glucagon, in high doses (over 1 mg), can cause rebound hypoglycemia due to a paradoxical increase in insulin secretion (97). Long-term non-surgical management of CHI using continuous subcutaneous glucagon infusion at a rate of 5-10 mcg/kg/hour in combination with octreotide have been reported (98,99).

**Frequent feeding:** Frequent feeding with high calorie carbohydrate feeds may reduce the frequency and severity of hypoglycaemic episodes. However, patients wth CHI, particularly those on diazoxide therapy usually have food aversion. Therefore a percutaneous gastrostomy is sometimes recommended to allow frequent (or continuous) feeding (100,101). Using complex carbohydrate such as uncooked cornstarch may decrease the hypoglycaemic episodes and improve fasting tolerance during a prolonged overnight fast in children over the age of one year.

### Long-term Management

A long-term management plan should be individualized for each patient and aim to normalize plasma glucose levels, provide an age-adjusted fasting tolerance and avoid neurological symptoms associated with hypoglycemia. Pharmacological therapy should be introduced one at a time to gauge the response and carefully monitored for side effects.

**Diazoxide:** Diazoxide, a K_ATP_ channel opener, is invaluable for managing many patients with CHI (1,32,96,102). Diazoxide is usually effective in all forms of CHI where the K_ATP_ channel function is intact but patients with recessive (and some dominant) K_ATP_ channel mutations do not respond to diazoxide (1). Diazoxide functions by binding to the SUR1 subunit of K_ATP_ channel. Thus, it requires a functionally intact K_ATP_ channel. Diazoxide responsiveness has been the key for molecular genetics analysis, differential diagnosis and management strategies of CHI. In diazoxide unresponsive CHI cases, urgent genetic analysis for ABCC8/KCNJ11 and ^18^F-DOPA-PET/CT scan are indicated to identify those patients who could have the focal form of CHI. In a recent study, diazoxide responsive patients with CHI who carry paternally inherited ABCC8 or KCNJ11 mutations have been reported and thus it was suggested that these patients should also undergo scanning with ^18^F-DOPA PET/CT (103).

The initial dose of diazoxide is 5 mg/kg/day, in three divided doses which can be increased up to a maximum dose of 15-20 mg/kg/day (104). The citeria for diazoxide responsiveness include an age adjusted fasting tolerance, able to maintain normoglycaemia and have a normal feeding plan. The most severe side effect that limits and requires treatment withdrawal is fluid retention, cardiac failure and the associated electrolyte imbalance. Diazoxide induced pulmonary hypertension is another life-threatening side effect which requires treatment withdrawal and therefore the FDA has issued a drug safety communication warning (105,106,107,108). In the newborn period a thiazide diuretic (such as chlorothiazide 7-10 mg/kg/day in two divided doses), is usually administered with diazoxide to prevent fluid retention. Other side effects of diazoxide therapy are described in Table 3 (33,102,109).

**Octreotide:** Octreotide, is an eight amino acid, synthetic, long-acting somatostatin analogue that inhibits insulin secretion by binding to somatostatin receptors 2 and 5 (SSTR2 and SSTR5) (110). Activation of SSTR5 decreases insulin gene promoter activity, inhibits calcium mobilization and acetylcholine activity (111). Somatostatin also inhibits the K_ATP_ channel which results in reduced insulin secretion (96). The recommended initial dose of octreotide is 5 μg/kg/day given by subcutaneous injections (or as a continuous infusion) at 6-8h intervals with a maximum dose of 30-35 μg/kg/day. Long-term, continuous, subcutaneous octreotide infusion with an insulin pump has also been reported as a feasible alternative to surgery for patients with monoallelic K_ATP_-channel mutations (112). The first response to octreotide administration is usually hyperglycaemia followed by a blunted effect after 48 hours (tachyphylaxis). Thus dose adjustment may be required (32,113,114). Although various side effects have been reported in case reports, in a study evalutaing the long-term safety and efficacy of octreotide in a large series of CHI patients, it was found to be a safe and effective treatment for diazoxide unresponsive CHI patients (102,115,116,117,118,119,120,121,122,123) (Table 3). The effect of octreotide on linear growth have been found clinically insignificant (102,117,123). In a recent clinical trial, monitoring the serum concentration of octreotide is recommended for dose titration, in order to avoid paradoxically diminished effectiveness and to reduce the side effects, thereby achieving optimal doses for highest efficacy and safety (123).

**Long-acting somatostatin analogs:** As conventional octreotide therapy requires multidose daily injections, this causes a burden to the patients and family, reduces adherence to the treatment and impacts negatively on quality of life (QoL). Monthly injection of long-acting somatostatin analogs have been described as an effective option in the management of CHI. Long-acting octreotide release (LAR) is formulated with biodegradable microspheres (124). This formulation increases the half-life with the advantage of being administrated every 28 days. Lanreotide is also a synthetic octapeptide and it can be adminstered every 28 days. LAR-octreotide and lanreotide have been used successfully in children with CHI, even in early infancy (102,125,126,127,128,129,130,131). Using LAR once every four weeks increases the treatment adherence and improves QoL (125).

**Nifedipine:** As the voltage gated calcium channel plays a key role in insulin secretion from the pancreatic β-cell, nifedipine, an L-type calcium channel blocker, has been used in the treatment of CHI (132). There have been several case reports demonstrating the effectiveness of Nifedipine in CHI patients. (133,134,135,136,137,138). In a recent study exclusively investigating long-term use of nifedipine in eleven CHI cases with ABCC8 mutations, none of patients showed any improvement in glycemic control and patients continued to have hypoglycemic episodes (139). This suggests that mutations in the K_ATP_ channel genes might render the L-type calcium channel ineffective to therapy with nifedipine (139) The recommended dose is 0.25-2.5 mg/kg/day divided into 2-3 doses (96). Hypotension is an uncommon side-effect (96), especially at doses above 0.5 mg/kg/day (134) (Table 3).

### New and Potential Future Therapies

Although our knowledge of the molecular basis of CHI has advanced, there are still challenges in managing patients who are diazoxide unresponsive. Most patients with diffuse CHI who are diazoxide unresponsove will typically require a near total pancreatectomy. In some patients, despite this major surgery, hypoglycemia persisted. Thus novel medical treatments are required to try and avoid a near total pancreatectomy which is not always curative.

**Sirolimus:** Sirolimus, an immunosuppressive agent with an anti-proliferative ability, inhibits the mammalian target of rapamycin (mTOR), a serine/threonine kinase (140). mTOR regulates cellular growth by stimulating protein synthesis and increasing mRNA translation initiation (141,142). The mechanism of action for mTOR inhibitors in CHI has not been fully elucidated. However, it is reported that there is constitutive activation and overexpression of p-mTOR on the plasmalemmal aspect of the acinar cells and activation on the plasmalemmal aspect of the ductal cells in the diffuse variant of CHI (143). Recently, another mechanism has been proposed; that sirolimus causes depletion of intracellular Ca^2+^ stores and alters mitochondrial activity, eventually leading to decreased insulin release (140). Upregulation of mTOR leads to increased insulin release from the pancreatic β-cells (144). Conversely, mTOR inhibition with rapamycin reduces insulin secretion as well as β-cell growth (145). Sirolimus can also enhance β-cell apoptosis and insulin resistance by reducing islet mass, insulin content and insulin sensitivity (140). mTOR also inhibits peroxisome proliferators-activated receptor-γ activity thereby affecting ketone body synthesis (146).

Sirolimus has been reported to be an effective and safe drug for severe, diazoxide unresponsive, diffuse CHI with no major side effects (147). Following the first report, significant numbers of cases have been reported (148,149,150,151,152,153,154). As sirolimus has potentially adverse effects (perhaps related to dose) arising from its immunosuppressive effects, measurement of the blood levels is vitally important for reaching an optimal therapeutic level. The most commonly reported adverse effects are stomatitis, increased risk of infection, immunosuppression, renal dysfunction, fatigue, pneumonitis and increased serum aminotransferase or lipid levels (155).

In a recent report evaluating the efficacy of sirolimus in 10 patients with diazoxide unresponsive CHI, mTOR inhibition has shown to be effective in only three patients (30%) with certain side effects (156). In addition, pancreatic tissue from two patients who did not respond to sirolimus showed no reduction in β-cell proliferation. Therefore it was claimed that inhibition of mTOR signaling does not down-regulate the β-cell proliferation in patients with CHI (156). Thus furthur studies, ideally in the form of clinical trails are required to assess the efficacy of mTOR inhibitors in CHI patients.

### Glucagon-like peptide-1 Receptor Antagonist: Exendin-(9-39)

GLP-1 is an incretin hormone produced in enteroendocrine L-cells of the intestine in response to ingested nutrients (157). GLP-1 enhances insulin secretion by binding to a guanine nucleotide binding protein-coupled receptor (158), resulting in the activation of adenylate cyclase and generation of cAMP (159). GLP-1 stimulates insulin secretion by both protein kinase A-dependent and -independent mechanisms (160) and also inhibits glucagon secretion, hepatic glucose production, gastric emptying and appetite.

Exendin-(9-39) is a specific GLP-1 receptor antagonist in mice and humans (161,162). In Sur-1 knock-out mice it was shown that Exendin-(9-39) decreases cAMP levels and inhibits insulin secretion thereby raising fasting plasma glucose levels (163). Another study demonstrated that exendin-(9-39) prevents hypoglycemia and maintains normoglycemia during a prolonged fast in individuals with K_ATP_ mutations (164). These promising results point to the GLP-1 receptor as a therapeutic target for K_ATP_ mutations. More recently, in the first population pharmacokinetic model of exendin-(9-39) in patients with CHI, the maximum recommended starting dose was determined to be 27 mg/kg/day, intravenously (165). This result informs the optimal dosing regimen for future clinical trials in neonates with CHI.

**Ketogenic diet:** CHI typically deprives the brain of both its main and alternative energy sources, being glucose and ketone bodies respectively. During the suckling period, ketone bodies constitute the main energy substrate for the brain. However, in the adult brain glucose is the main energy source (166). An increase in the ketone body concentration increases their oxidation rate in the brain (167,168). Thus, ketogenic diets have been used as an adjunctive therapeutic option in refractory epilepsy and in experimental models of ischemia and excitotoxicity (169). HH induces severe neuroglycopenia and also inhibits gluconeogenesis, glycogenolysis, lipolysis and, eventually, fatty acid oxidation which results in suppressed ketone body synthesis. This makes the brain more vulnerable to the neurological insult of hypoglycaemia. Maiorana et al (170) reported a trial ketogenic diet administered to a child with CHI due to a spontaneous GCK activating mutation and recurring hypoglycaemic episodes, despite medical therapy. After the first six months, the patient was free of epileptic seizures, with normalization of EEG and showed a marked recovery in psychological development and QoL (170). Although this treatment requires further investigation these initial findings suggest that a ketogenic diet could have a neuroprotective effect in selected cases of CHI.

### Histologic Subtypes of Congenital Hyperinsulinaemic Hypoglycaemia

**Figure 2:**
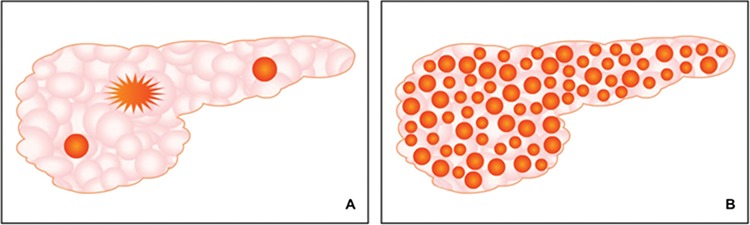
A schematic representation of focal and diffuse congenital hyperinsulinism. In the focal disease (A), the β-cell hyperplasia is limited to a certain are of the pancreas gland with a superficial or deep localization or invades as a tentacle shape. In the diffuse disease (B) there is a global β-cell hyperplasia throughout the whole panreas

**Figure 3:**
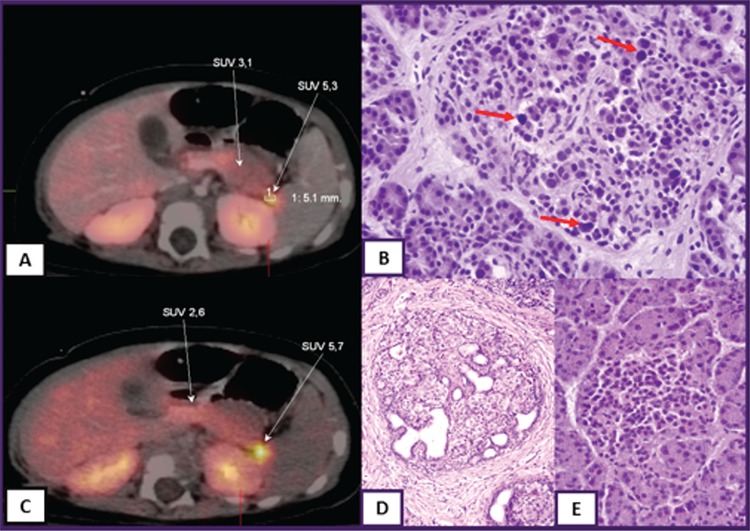
^18^F-fluoro-L-dihydroxyphenylalanine (^18^F-DOPA)-positron emission tomography/computed tomography scan images of focal congenital hyperinsulinism (A and C), histological figure of diffuse (B) and focal (D) disease and normal pancreas islet cell (E). Standardized uptake value (SUV) 5.3 and SUV 5.7 indicate focal uptake of ^18^F-DOPA, red arrows show large nuclei of β-cell in diffuse disease

In terms of histology, there are three forms of CHI; focal, diffuse, and atypical disease (Figure 2). In focal CHI the abnormal pancreatic β-cells are localised to a specific region of the pancreas. Focal pancreatic lesions are generally 2-10 mm in size and appear as small regions of islet adenomatosis (nodular hyperplasia of islet-like cell clusters, including ductuloinsular complexes, Figure 3) (33). Islet cells in the lesion have large cytoplasm with dispersed abnormal nuclei of irregular shape (171).

Focal disease is mostly sporadic and is associated with a paternally inherited K_ATP_ channel mutations and the loss of maternal heterozygosity for 11p in the focal area (172). This in turn induces the expression of insulin-like growth factor 2, inhibits the tumor suppressor genes H19 and cyclin-dependent kinase inhibitor 1C and leads to β-cell proliferation (173). ^18^F-DOPA-PET scanning is currently the only diagnostic imaging tool to accurately localize focal lesions (174). Pancreatic islets are able to uptake L-DOPA and convert it to dopamine through DOPA decarboxylase. The uptake of the positron emitting tracer ^18^F-DOPA-PET is increased in β-cells with a high rate of insulin synthesis and secretion compared to unaffected areas (Figure 3). The sensitivity for detecting focal lesions varies between 88 and 94% with an accuracy of 100% (175). In a recent study ^18^F-DOPA-PET/CT was found to be superior in localizing focal lesions compared to imaging with ^68^Ga-DOTANOC PET/CT (176). Patients with focal CHI are usually unressponsive to medical therapy and require a surgical lesionectomy.

Diffuse disease accounts for about 60% of all CHI cases and affects all the β-cells of the pancreas. Morphology of the islets of Langerhans typically show the presence of β-cells with abnormally large nuclei (Figure 3) (177). Patients with diffuse CHI mostly have either a homozygous recessive or a compound heterozygous mutations in K_ATP_ channel genes (8). Patients are usually unresponsive to medical therapy and require a near-total pancreatectomy (95-98 % removal).

In some cases pancreatic histology does not fit the typical focal or diffuse appearance and therefore atypical forms of CHI have been described (178,179,180). In atypical forms some islets show signs of hyperplasia interpersed with histologically normal looking islets. Some patients with CHI have morphological mosaicism including coexistence of two types of islet; large islets with cytoplasm-rich cells and occasional enlarged nuclei and shrunken islets with cells exhibiting little cytoplasm and small nuclei (173).

### Surgical Therapy

Differentiation of the histological subtypes is essential for successful surgical outcome. Recent advances in the molecular genetics of CHI and imaging with ^18^F-DOPA-PET/CT have changed the management of patients, particularly those with focal disease (177). In diffuse disease there is uptake of ^18^F-DOPA throughout the pancreas on the PET/CT scan whereas in focal forms there is limited uptake of ^18^F-DOPA in a localised region of the pancreas. Once this focal lesion is localised on the PET/CT it is possible to surgically remove the lesion and cure the patient of hypoglycemia (Figure 3) (181,182). Intraoperative frozen sections are important as these can both confirm the histological diagnosis and to determine the margin of resection (183).

**Surgery for diffuse and atypical disease**: Patients with diffuse and atypical disease usually require extensive surgery (subtotal- or near-total pancreatectomy). This procedure caries a high risk of developing pancreatic exocrine insufficiency and diabetes which requires life-long pancreatic enzyme replacement and insulin therapy (7,184,185,186,187). In near-total pancreatectomy, the tail, body, uncinate process and part of the pancreatic head are resected, leaving a rim of pancreatic tissue surrounding the common bile duct and along the duodenum (7). However, despite extensive resection (95-98% of pancreatic tissue) some children continue to have HH (185). Diabetes can develop immediately after surgery or later during follow-up (184). Therefore, patients who undergo surgical resection should be monitored for glucose metabolism and diabetes (184,185,186,187).

### Follow up and Outcome of Congenital Hyperinsulinism

The management of patients with severe CHI is challenging and requires a multi-disciplinary team approach which should include clinicians, surgeons, specialized pathologists, geneticists, nurse specialists and dietitians. In studies evaluating the long-term outcome of patients with HH, a high frequency of neurodevelopmental retardation and various neurological disorders, including epilepsy and microcephaly, have been reported (187,188,189). Severity of the disease (based on maximal diazoxide dose) and early presentation (<7 days following birth) were associated with abnormal neurodevelopment, while gender, underlying genetic etiology or the histopathological form of HH were not related to the neurological outcome (189). In a recent study evaluating long-term neurodevelopmental outcome of 60 patients with CHI, just under two fifths of cases were shown to be affected with motor deficits (38.6%) followed by speech problems (26.9%), cognitive deficits (15.8%) and social-emotional problems (9.4%), with no correlation between outcome and genetic background (190). Therefore, neurological development should be closely followed up, regardless of the underlying etiology and histopathological type.

**Figure 4:**
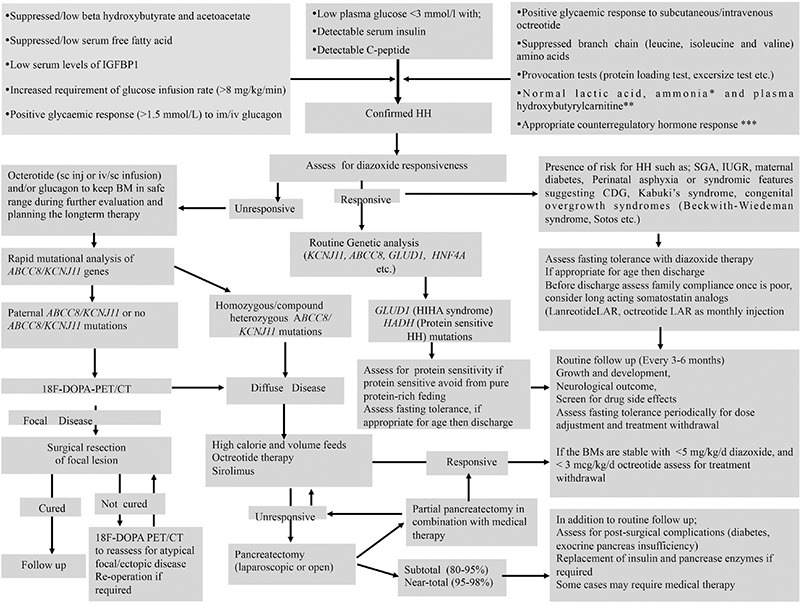
An algorithm for the diagnosis and management of hyperinsulinaemic hypoglycaemia HH: hyperinsulinaemic hypoglycaemia, IGFBP-1: insulin growth factor binding protein-1, HNF4A: hepatocyte nuclear factor 4A, ABCC8: ATP binding cassette subfamily C member 8, KCNJ11: potassium voltage-gated channel subfamily J member 11, GLUD1: glutamate dehydrogenase 1, HADH: hydroxyacyl-CoA dehydrogenase, LAR: long-acting release, IUGR: intrauterine growth restriction, CDG: congenital disorders of glycosylation, SGA: small for gestational age, ^18^F-DOPA-PET/CT: ^18^F-fluoro-L-dihydroxyphenylalanine-positron emission tomography/computed tomography

Figure 4 outlines management and follow-up of patients with congenital HH.

### Conclusions and Future Directions

CHI is the most common cause of severe hypoglycaemia in the newborn and childhood period. The molecular basis of CHI involves defects in key genes (ABCC8, KCNJ11, GLUD1, GCK, HADH, SLC16A1, HNF1A, HNF4A, UCP2, HK1, PGM1, PMM2 and FOXA2) which regulate insulin secretion. Rapid genetic analysis, imaging with ^18^F-DOPA-PET/CT scan, potential new medical therapies and development in surgical techniques have improved the management and outcome of the disease. Further research is needed to identify the underlying molecular basis of CHI, especially in patients who are diazoxide responsve. Novel, routinely available imaging techniques should be developed so that patients all over the world can have access to these facilities.

## Figures and Tables

**Table 1:**
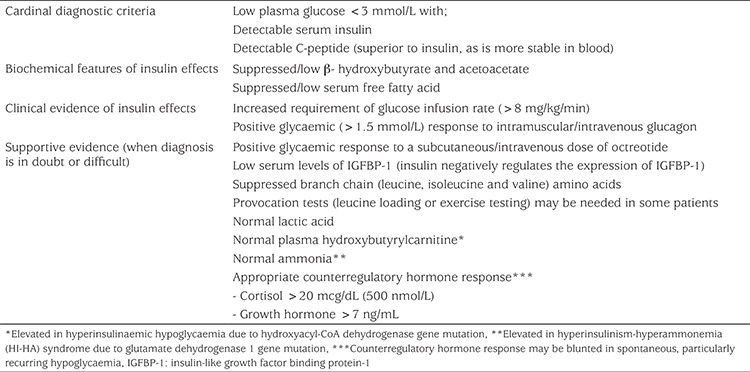
Diagnostic criteria for hyperinsulinaemic hypoglycaemia

**Table 2:**
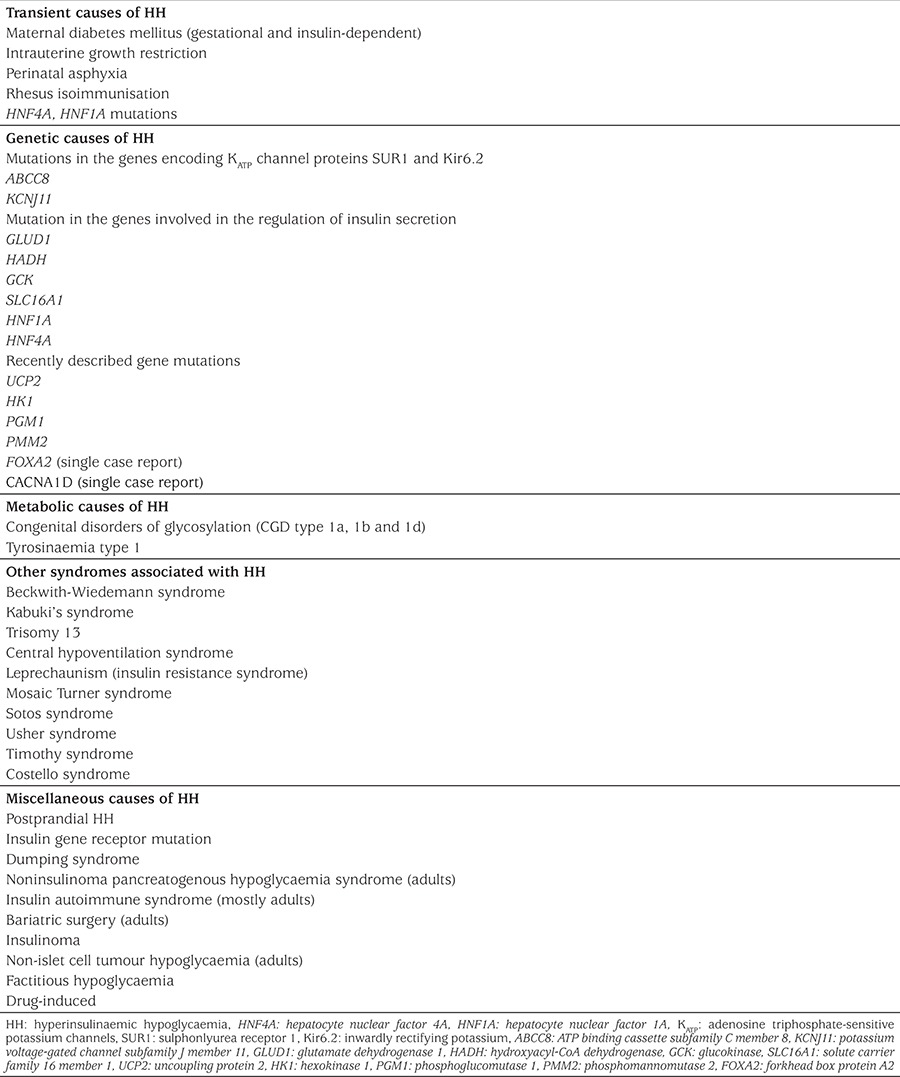
Transient and permanent causes of hyperinsulinaemic hypoglycaemia

**Table 3:**
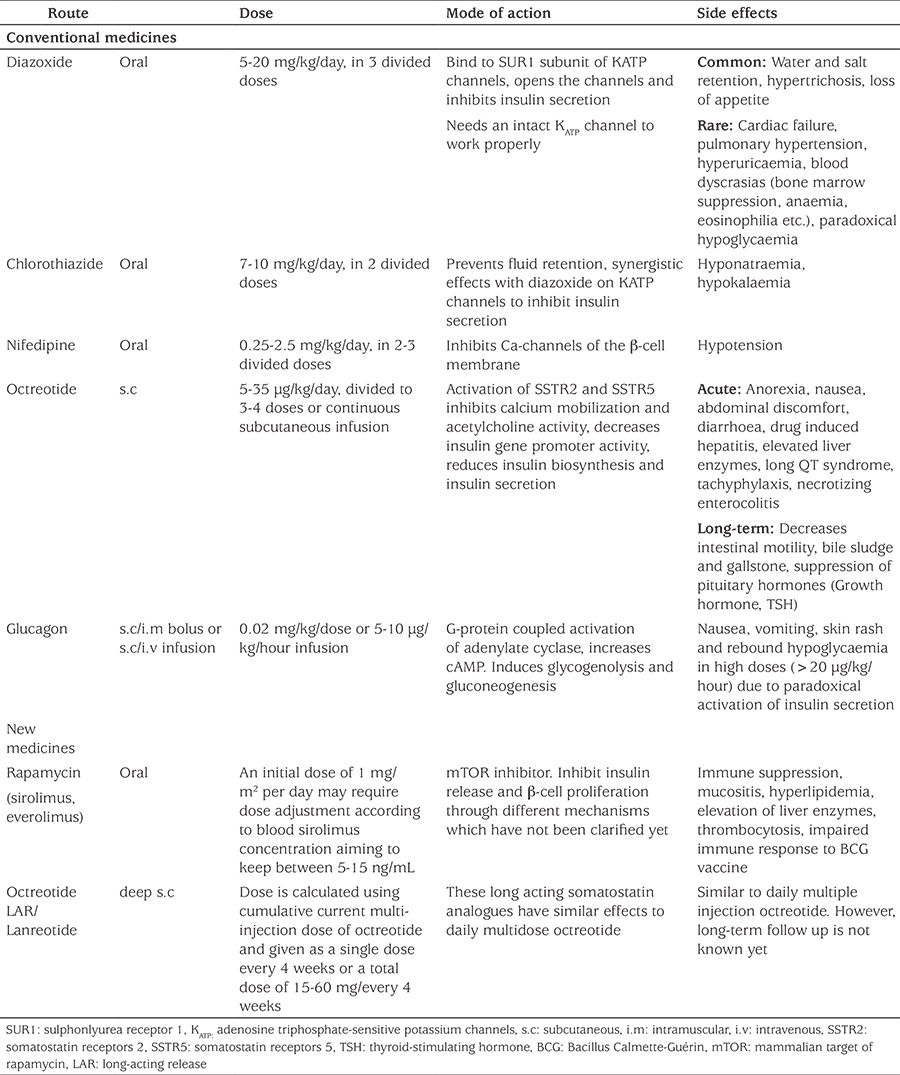
Drugs for medical therapy of hyperinsulinaemic hypoglycaemia
